# Out-patient triple chronotherapy for the rapid treatment and maintenance of response in depression: feasibility and pilot randomised controlled trial

**DOI:** 10.1192/bjo.2021.1044

**Published:** 2021-11-24

**Authors:** David Veale, Marc Serfaty, Clara Humpston, Andriani Papageorgiou, Sarah Markham, John Hodsoll, Allan H. Young

**Affiliations:** Institute of Psychiatry, Psychology and Neuroscience, King's College London & South London and Maudsley NHS Foundation Trust, London, UK; Division of Psychiatry, University College London, UK; and The Priory Hospital North London, UK; Institute of Psychiatry, Psychology and Neuroscience, King's College London, UK; and Institute for Mental Health, School of Psychology, University of Birmingham, UK; Institute of Psychiatry, Psychology and Neuroscience, King's College London, UK; Institute of Psychiatry, Psychology and Neuroscience, King's College London, UK; Institute of Psychiatry, Psychology and Neuroscience, King's College London, UK; Department of Psychological Medicine, Institute of Psychiatry, Psychology and Neuroscience, King's College London & South London and Maudsley NHS Foundation Trust, London, UK

**Keywords:** Depressive disorders, randomised controlled trial, out-patient treatment, outcome studies, rehabilitation

## Abstract

**Background:**

Triple chronotherapy (sleep deprivation for 36 h, followed by 4 days of advancing the time of sleep and daily morning bright-light therapy for 6 months) has demonstrated benefits for the rapid treatment of depressive symptoms in four small controlled trials of in-patients.

**Aims:**

To test the feasibility of recruitment and delivery of triple chronotherapy for out-patients with depression (ISRCTN17706836; NCT03405493).

**Method:**

In a single-blind trial, 82 participants were randomised to triple chronotherapy or a control intervention. The primary outcome was the number of participants recruited per month and adherence to the protocol. Secondary outcomes included the 6-item Hamilton Rating Scale for Depression (HRSD-6) at 1 week. Timings of observer ratings were baseline and 1, 2, 4, 8 and 26 weeks after randomisation.

**Results:**

The triple chronotherapy group stayed awake for the planned 36 h and 89.9% adhered to the plan of phase advance of their sleep over the following 4 days. We achieved our recruitment target (60 participants completed the trial within 13 months). There were no reported adverse side-effects. We found a significant difference between the groups by intention-to-treat analysis for the HRSD-6 at weeks 1, 8 and 26. There was a large effect size of Cohen's *d* = 0.8 on HRSD-6 score at week 1, increasing to *d* = 1.30 at week 26. A response (≥50% reduction in symptoms) was achieved by 33.3% in the triple chronotherapy group and 16.2% in the control group. This stayed relatively steady until week 26 (35.9 *v*. 13.9%).

**Conclusions:**

Triple chronotherapy produced a significant and rapid benefit after 1 week in out-patients with depression that was sustained at 26 weeks. Cost-effectiveness trials with a larger clinical sample are required.

There are many treatments for depression with modest efficacy and there is much need and scope for improvement. The effect size for antidepressants against placebo derived from meta-analyses is approximately Cohen's *d* = 0.3 at 8 weeks^1,2^ and for cognitive–behavioural therapy the effect size is *d* = 0.4 at 12 weeks.^3^ However, an identified problem is that the earliest response (defined as 50% reduction in symptoms) for an established treatment of depression such as antidepressant medication^2^ or a psychological therapy^3^ may not occur for at least 4–6 weeks. One of the most rapid antidepressants known since the 1970s is a night of total sleep deprivation.^4^ A meta-analysis of 9 randomised controlled trials (RCTs) and 26 case series on total sleep deprivation for depression found that about 50% of patients recovered within 24 h.^5^ However, of those who recovered, about 85% relapsed following a night's sleep, with a diminishing effect if naps were taken during the period of sleep deprivation.^5,6^ Triple chronotherapy has been developed to sustain this rapid response through administering total sleep deprivation for 36 h, followed by 4 days of advancing the time of sleep, together with daily morning bright-light therapy for 6 months. The rationale is that depression (in some individuals) is associated with circadian rhythm disturbances.^7,8^ The theory is that triple chronotherapy resynchronises the circadian rhythms, possibly by acting through multisystem mechanisms by regulating neurotransmitters and hormones.^7^ D'Agostino et al^9^ published a narrative review and Humpston et al^10^ conducted a meta-analysis of four RCTs (range *n* = 14–75 patients) and 12 case series (*n* = 504 patients) of triple chronotherapy for both unipolar and bipolar depression, almost entirely in in-patients. This showed that for RCTs, triple chronotherapy was favoured at 1 week (Hedge's *g* = 0.62) and at 8 weeks (*g* = 0.35) compared with active control treatments such as antidepressants or exercise. For the case series, large effect sizes pre–post within the group were found at 1 week (*g* = 1.79) with a weighted mean response rate of 61.6%, suggesting that triple chronotherapy appears promising for the treatment of depressive disorder in in-patients. On the basis of these results, the aim of the current study was to conduct a feasibility and pilot RCT of triple chronotherapy for unipolar depression in out-patients.

## Method

### Trial design

A randomised parallel-group single-blind design comparing the addition of triple chronotherapy or a control sleep intervention to standard care was used. The protocol was first written in July 2017 and was modified in January 2018. Blue-light blocking glasses were added to the protocol before the trial began. No previous piloting work was undertaken in 2017 and no changes were made to the protocol after the trial began.

#### Participants’ eligibility criteria

Inclusion criteria were an ICD-10 diagnosis of depressive episode (ICD-10 F32) or recurrent depressive disorder (F33); score of 8 or more on the 6-item Hamilton Rating Scale for Depression (HRSD-6) (also abbreviated as HAM-D6),^11^ aged 18–65 years and capable of giving informed consent.

Exclusion criteria were a current diagnosis of seasonal affective disorder, anorexia nervosa or bulimia nervosa, obsessive–compulsive or related disorder, or post-traumatic stress disorder or emotionally unstable personality disorder considered to be the main problem; history of schizophrenia, schizoaffective disorder or bipolar disorder; severe cognitive impairment or organic brain disorder; history of stimulant or hallucinogenic misuse, alcohol or substance misuse or dependence in past 3 months; duration of depression more than 2 years; significant risk of suicide that requires hospital admission; blindness or visual impairment affecting both eyes (which diminishes or blocks light therapy); history of epilepsy, as this may lower seizure threshold through sleep deprivation; untreated sleep disorder such as obstructive sleep apnoea or narcolepsy; use of photo-sensitising drugs; current night-shift work; and non-English speaker.

The use of antidepressant medication did not exclude a participant as long as the dose had been stable for 6 weeks and there were no plans to alter the medication during the course of the trial. Equally, the use of psychotherapy did not exclude a patient.

#### Settings and locations

Participants were mainly recruited from primary care (Improving Access to Psychological Therapies, IAPT) services in South London and Maudsley NHS Foundation Trust (77.5%). Other sources included self-referrals (21.4%) and general practitioner referrals (1.1%). No referrals were received from secondary or tertiary care.

### Interventions

All eligible participants were randomised to receive either triple chronotherapy or a control sleep intervention.

#### Triple chronotherapy

The timetable for triple chronotherapy is shown graphically in supplementary Fig 1, available online at http://dx.doi.org/10.1192/bjo.2021.1044. It consisted of the following.

- Total sleep deprivation for 36 hours. On day 1 (usually a Friday), participants, in small groups, were helped to stay awake at night by an occupational therapist.
- Phase advance of sleep over 4 days. Phase advance began after the first night of sleep deprivation, when the participants left the hospital at about 08.00 h and were asked to go to bed earlier, at about 17.00 h, and to rise at about 01.00 h. Their sleep and wake up times were then shifted 2 h later on each of the following 3 days until they attained their usual bedtime again (about 23.00 h). During the period of phase advance, they were given amber (blue-blocking) glasses manufactured by SomniLight to wear 3 h before going to bed. This was designed to encourage release of melatonin and prevent a phase delay of sleep by evening light exposure.
- Daily bright-light therapy was given on day 2 and continued for 6 months. The time of the light therapy was optimised in the morning in accordance with each patient's chronotype and pattern of sleep, as determined by the Morningness–Eveningness Questionnaire.^12^ A person's chronotype is their internal circadian rhythm or body clock, which influences timing of the cycle of sleep and activity in a 24 h period. Participants were asked to sit about 1 foot (30 cm) away from a bright-light box that emitted 10 000 lux (Carex Day-Light Classic®). It was positioned slightly above the head and pointing downwards at 45°. They were required to sit facing the light box (simply having a light box in the room does not ensure that the person receives adequate light intensity at eye level). The white light incorporates blue–green light in the 540 nm spectrum, which is known to suppress melatonin and to ‘phase advance’ its secretion if light is given in the morning. Patients were free to have breakfast, read or use a computer while facing the light. Treatment normally lasted 30 min and continued daily for 6 months. Further details on the practice of chronotherapy can be found in Wirz-Justice et al.^13^

#### Control intervention

As a control for the triple chronotherapy, participants were given psychoeducation and written information on sleep hygiene and getting a good night's sleep, with the opportunity to ask questions. They were also given amber light (an amber lamp (SomniLight Amber Light, discontinued) manufactured by SomniLight) daily for 1 week in the morning. The rationale was that amber light (peaking at 592 nm wavelength and 300 lux at 30 cm) does not suppress melatonin secretion or have a significant impact on the melatonin phase and it was given as a control for the bright-light therapy. Participants had the same instructions as for the bright-light therapy and optimisation of timing for the light in the morning.

### Outcomes

The primary outcome was the number of participants recruited per month and adherence to the protocol, with 1 week as the primary end-point. The secondary outcome measures were the masked (‘blind’) observer-rated 6-item Hamilton Rating Scale for Depression (HRSD-6)^11^ (total range 0–22: scores 0–4, no or minimal depression; 5–8, mild; 9–10, moderate; 11–13, severe; >13, very severe); the observer-rated Clinical Global Impression – Improvement scale;^14^ the Quick Inventory of Depressive Symptomatology – self-report version;^15^ the Brief Penn State Worry Questionnaire (5-item) and the Ruminative Response Scale (5-item);^16^ the Pittsburgh Sleep Quality Index;^17^ and the EuroQoL 5D.^18^ All measures were done at baseline and at 1, 2, 4, 8 and 26 weeks post-randomisation. Participants were given a commercial tracker (Fitbit^®^) for 1 week to collect activity and sleep data.

### Sample size

This was a feasibility and pilot study and therefore we wished to evaluate feasibility parameters such as the rate of recruitment, loss to follow-up and treatment adherence and to estimate the variability of the outcome. Thirty people equally randomised to each group completing a study is usually considered adequate for a feasibility and pilot study^19^ and allows informative 95% confidence intervals (CIs) to be estimated on feasibility parameters. Of the 173 individuals approached, 82 participants were randomised for 60 people to complete the study.

### Randomisation

The method of randomisation consisted of simple randomisation based on a constant allocation ratio of 1:1. It was stratified according to the season.

Randomisation was conducted by an independent research worker via the King's Clinical Trials Unit (which is registered with the UK Clinical Research Collaboration) using a web-based random sequence generator system. The same research worker concealed the sequence until he assigned participants to the interventions.

### Sources of bias

Participants recorded the credibility of the treatment and expectancy of benefit after they were allocated to a group.^20^ The use of antidepressant medication and whether they were receiving any counselling or psychotherapy was recorded at baseline.

### Masking

The research associate administering the observer-rated assessments was masked to group assignment. Participants were reminded at each time point the importance of not disclosing under any circumstances the treatment to which they were assigned. Participants were enrolled, screened and assessed by a different research worker from the one who organised the interventions for all participants. The research workers each had separate recording systems and different passwords to maintain masking.

### Analysis

Feasibility outcomes were presented as frequencies and proportions. We explored outcomes and our analysis presents means, 95% CI and effect sizes for the two intervention groups by time (at 1, 2, 4, 8 and 26 weeks). We conducted a linear mixed model analysis to determine the estimated means and standard errors of outcomes using the maximum likelihood estimation. The model uses all available data. If data are missing at random, model parameters will be unbiased. We also calculated a standardised effect size and confidence interval between the groups from all available data at each time point by dividing the estimated mean difference (from the mixed model) by the standard deviation of the measure at baseline. As per the rule of thumb for Cohen's *d*, standardised effect sizes were described as small (SES = 0.2), medium (SES = 0.5) or large (SES = 0.8) and effect sizes were presented with 95% CI. Where hypothesis tests were carried out, the criterion for significance was *P* < 0.05 and all tests were two-tailed. Data on all participants who started treatment were analysed according to their randomised groups (modified intention to treat). Participants who were randomised but did not commence treatment were included in a consideration of drivers of pre-treatment drop-out.

### Ethics

Written informed consent was obtained from all participants. The authors assert that all procedures contributing to this work complied with the ethical standards of the relevant national and institutional committees on human experimentation and with the Helsinki Declaration of 1975, as revised in 2008. All procedures involving human participants/patients were approved by the London Bromley Research Ethics Committee (reference 17/LO/1567). The trial is registered with Clinicaltrials.gov (NCT03405493) and the ISRCTN registry (ISRCTN17706836).

## Results

### Participant flow

**Fig. 1:**
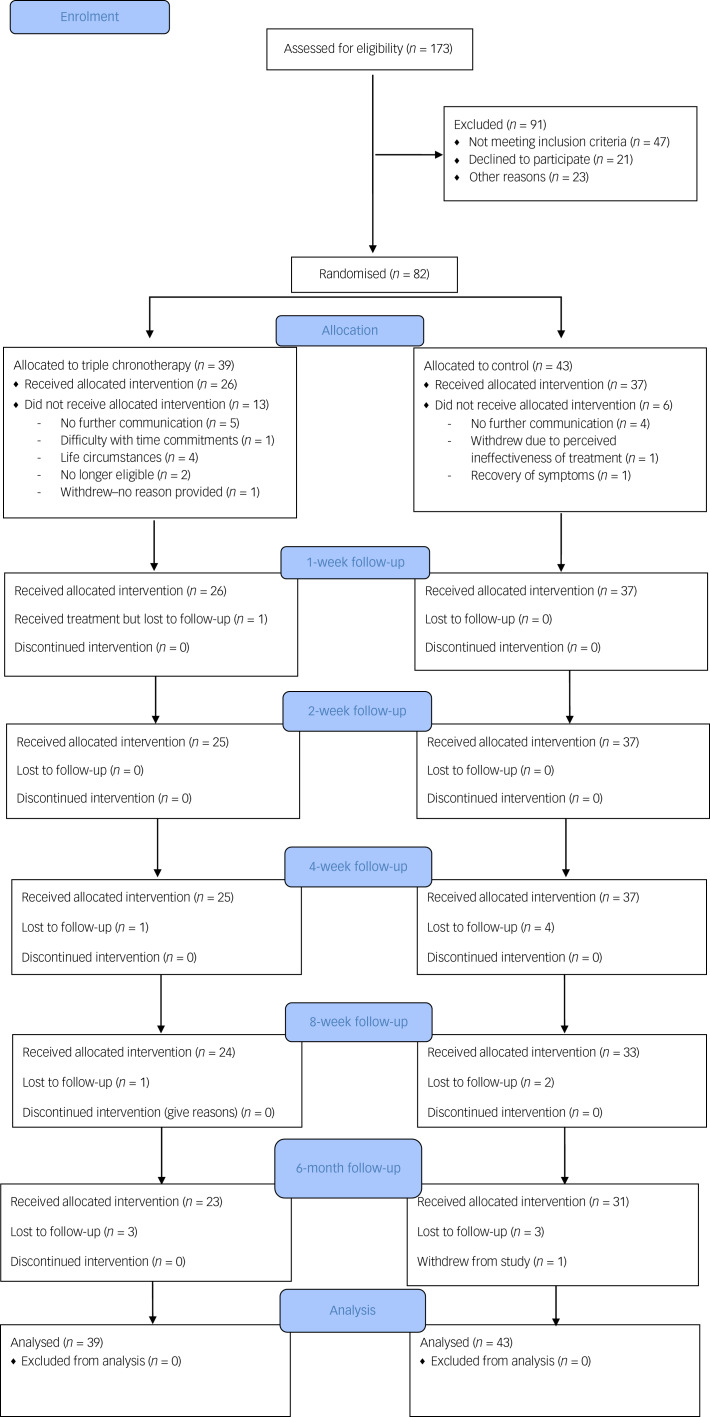
CONSORT flowchart.

A CONSORT flowchart is shown in Fig. 1. We achieved the planned rate of recruitment over 13 months, which was one of our primary outcomes.

### Recruitment

Participants were recruited between the 5 February 2018 and 22 March 2019. The final follow-up was on 13 September 2019. Inclusion rate into the study was less than 50% (screening 173 individuals for 82 to be randomised).

### Baseline data

Participant demographics are shown in Table 1. Of the 82 participants, 44 were not taking any medication; 38 were taking an antidepressant or mood stabiliser (citalopram, *n* = 13; escitalopram, *n* = 5; fluoxetine, *n* = 2; mirtazapine, *n* = 1; paroxetine, *n* = 1; sertraline, *n* = 14; quetiapine, *n* = 1; and lamotrigine, *n* = 1); 34 participants were receiving psychotherapy. There were no significant differences between the groups at baseline, including HRSD-6 scores.

**Table 1:** Demographic information

	Total (*n* = 82)	Triple chronotherapy (*n* = 39)	Control group (*n* = 43)
Age, years: mean (s.d.)	38.4 (13.3)	38.2 (12.5)		38.6 (14.2)	
Gender					
Female	45	18	46.2%	27	62.8%
Male	37	21	53.8%	16	37.2%
Relationship status					
Single	37	16	41.0%	21	48.8%
In relationship	45	23	59.0%	22	51.2%
Ethnicity					
White	65	32	82.0%	33	76.7%
Asian	4	3	7.7%	1	2.3%
Black	8	3	7.7%	5	11.7%
Mixed	4	–	–	4	9.3%
Other	1	1	2.6%	–	–
Education					
No qualifications	16	6	15.4%	10	23.3%
Diploma/A, O levels	5	4	10.3%	1	2.3%
College	4	2	5.1%	2	4.7%
Professional	20	5	12.8%	15	34.9%
Bachelor	16	10	25.6%	6	13.9%
Master's	19	11	28.2%	8	18.6%
Doctorate	2	1	2.6%	1	2.3%
Employment					
Student	6	3	7.7%	3	6.9%
Employed/self-employed	51	29	74.3%	22	51.2%
Homemaker	2	−	−	2	4.7%
Unemployed	13	4	10.3%	9	20.9%
Long-term sick	6	1	2.6%	5	11.6%
Retired	4	2	5.1%	2	4.7%
Current psychotherapy	34/82	17/39	43.6%	17/43	39.5%
Current antidepressant medication	38/82	16/39	41.0%	22/43	51.2%
Mean fluoxetine equivalent dose, mg	38.36	39.81		38.95	
MEQ score, mean (s.d.)	45.67 (10.17)	46.35 (10.17)		45.19 (11.80)	
Credibility rating (range 3–27), mean (s.d.)		17.9 (3.38)		18.1 (4.38)	
Expectancy rating for benefit (range 3–27), mean (s.d.)		15.0 (4.47)		14.0 (5.27)	

MEQ, Morningness–Eveningness Questionnaire.

### Pre-treatment drop-out

Eighty-two participants were recruited and randomised for this study and 23.2% (*n* = 19) did not commence either intervention after randomisation. The number who dropped out before the treatments commenced was *n* = 13 for the triple chronotherapy group and *n* = 6 for the control group (Fig. 1). Once participants had received the intervention the retention at 6-month follow-up was adequate in both the triple chronotherapy (77%, *n* = 20/26) and the control group (73%, *n* = 27/37). There were no significant differences between those who had at least 1 week of treatment and those who dropped out after randomisation in the frequency of taking antidepressant medication, receiving a psychological therapy, relationship status or employment status. There was a difference in baseline HRSD-6 scores, with those who dropped out (HRSD-6 score: mean 10.9, s.d. = 1.84) being significantly less depressed than completers (mean 11.89, s.d. = 1.66, *t* = −2.58, *P* < 0.05). There was also a significant difference in age (*t* = −2.5, *P* < 0.05), where those who dropped out were younger than those who received any treatment (mean 31.9 years, s.d. = 13.0 and mean 40.4 years, s.d. = 12.9 respectively), and gender (χ^2^(82) = 5.79, *P* < 0.05), as those who dropped out were biased towards more females (*n* = 15) than males (*n* = 4) in comparison with those who received any treatment (*n* = 30 females; *n* = 33 males). Of the 82 randomised, 26 received triple chronotherapy and 37 received the sleep control for at least 1 week.

### Missing outcome data for treatment starters

Frequency of missing data for HRSD-6 ratings was low in both groups up to week 8 (see supplementary Table 4), at 14% overall. At week 26, almost a quarter of participants had missing ratings (*n* = 6, 23%). Logistic regression with missingness as outcome showed no significant differences in rates of missingness between groups at weeks 8 and 26.

### Adherence (primary outcome)

All participants in the triple chronotherapy group adhered to the sleep deprivation. They were supervised by an occupational therapist to stay up at night without napping. In terms of acceptability of the intervention, adherence to triple chronotherapy was estimated by examining sleep diaries, which were submitted by 18 of the 25 participants (72%) for the first week after sleep deprivation. None reported going to sleep before 17.00 on the first day and only 2 participants out of the 18 (11.1%) were judged to be not adhering to the plan of phase advance. For the remainder in the 18, the planned time of sleep onset was occasionally advanced or delayed by up to 1 h against the plan. We collected 25 diaries from the 37 participants in the control group (67.7%) for the same period: 15 out of the 25 (60%) got up fairly consistently by 08.00 h in accordance with the sleep hygiene programme.

### Secondary outcomes

Table 2 gives the results of the linear mixed model analysis of secondary outcomes. Standardised effect sizes showed moderate to large differences between groups from week 1 onwards, and effect sizes were above 1 for weeks 8 and 26 (and statistically significant at weeks 1, 8 and 26). The raw scores are provided in supplementary Table 3. This was replicated at the same time points for the self-report measure on depression (Quick Inventory of Depressive Symptomatology, QIDS-SR). Effect sizes for the differences between two groups were more modest for the Penn State Worry Questionnaire (PSWQ) until week 8 and the effect size for the Ruminative Response Scale (RRS) and EuroQoL 5D (EQ-5D) were only small until week 26, at which point the large difference was statistically significant.

**Table 2:** Estimated mean group differences across time by intention-to-treat analysis

	Week 1	Week 2	Week 4	Week 8	Week 26
HRSD-6 (depression)					
Mean (95% CI)	1.87 (0.3 to 3.44)	1.47 (−0.09 to 3.03)	1.44 (−0.14 to 3.02)	3.4 (1.8 to 4.99)	4.01 (2.08 to 5.94)
SES (95% CI)	0.86 (0.14 to 1.58)	0.68 (−0.04 to 1.4)	0.66 (−0.07 to 1.39)	1.56 (0.83 to 2.3)	1.84 (0.96 to 2.73)
*t* (d.f.)	2.3 (110.7)	1.9 (114.9)	1.8 (128.5)	4.2 (135.4)	4.1 (54.2)
*P*	0.021	0.067	0.077	0.001	0.001
QIDS-SR (depression)					
Mean (95% CI)	3.02 (0.78 to 5.26)	1.18 (−1.05 to 3.41)	1.9 (−0.33 to 4.12)	3.73 (1.51 to 5.95)	4.47 (1.93 to 7)
SES (95% CI)	1.39 (0.36 to 2.42)	0.54 (−0.48 to 1.57)	0.87 (−0.15 to 1.9)	1.72 (0.69 to 2.74)	2.06 (0.89 to 3.22)
*t* (d.f.)	2.6 (105.8)	1 (110.8)	1.7 (119.8)	3.3 (128)	3.5 (49.4)
*P*	0.010	0.303	0.097	0.001	0.001
Brief PSWQ (worry)					
Mean (95% CI)	1.43 (−0.36 to 3.22)	1.17 (−0.61 to 2.95)	1.25 (−0.52 to 3.01)	2.27 (0.51 to 4.03)	2.88 (0.69 to 5.07)
SES (95% CI)	0.34 (−0.08 to 0.76)	0.28 (−0.14 to 0.7)	0.29 (−0.12 to 0.71)	0.54 (0.12 to 0.95)	0.68 (0.16 to 1.2)
*t* (d.f.)	1.6 (88.6)	1.3 (92.1)	1.4 (97.3)	2.5 (100.9)	2.6 (52.8)
*P*	0.120	0.200	0.169	0.013	0.013
Brief RRS (rumination)					
Mean (95% CI)	1.44 (0.01 to 2.87)	0.63 (−0.79 to 2.06)	0.49 (−0.93 to 1.91)	0.71 (−0.72 to 2.14)	2.25 (0.43 to 4.08)
SES (95% CI)	0.53 (0 to 1.05)	0.23 (−0.29 to 0.75)	0.18 (−0.34 to 0.7)	0.26 (−0.26 to 0.78)	0.83 (0.16 to 1.5)
*t* (d.f.)	2 (92.8)	0.9 (97.1)	0.7 (103.7)	1 (107.9)	2.4 (49.1)
*P*	0.051	0.387	0.497	0.334	0.019
PSQI (sleep)					
Mean (95% CI)	1.38 (−0.21 to 2.97)	2.2 (0.61 to 3.78)	2.36 (0.78 to 3.93)	2.35 (0.77 to 3.93)	1.92 (0.09 to 3.75)
SES (95% CI)	0.4 (−0.06 to 0.87)	0.64 (0.18 to 1.1)	0.69 (0.23 to 1.15)	0.68 (0.22 to 1.14)	0.56 (0.03 to 1.09)
*t* (d.f.)	1.7 (85.7)	2.7 (88.3)	2.9 (93.4)	2.9 (97.2)	2.1 (52.6)
*P*	0.093	0.008	0.004	0.004	0.045
EQ-5D (quality of life)					
Mean (95% CI)	−3.87 (−11.81 to 4.07)	−6.74 (−14.65 to 1.17)	−3.9 (−11.77 to 3.96)	−4.97 (−12.83 to 2.9)	−14.03 (−23.34 to −4.72)
SES (95% CI)	−0.17 (−0.52 to 0.18)	−0.3 (−0.64 to 0.05)	−0.17 (−0.52 to 0.17)	−0.22 (−0.56 to 0.13)	−0.62 (−1.03 to −0.21)
*t* (d.f.)	−1 (145)	−1.7 (154.8)	−1 (171.3)	−1.2 (186.2)	−3 (49.2)
*P*	0.341	0.097	0.332	0.217	0.005

HRSD-6, 6-item Hamilton Rating Scale for Depression; QIDS-SR, Quick Inventory of Depressive Symptomatology self-report version; PSWQ, Penn State Worry Questionnaire; RRS, Ruminative Response Scale; PSQI, Pittsburgh Sleep Quality Index; SES, standardised effect size; EQ-5D, EuroQoL 5D.

Response was defined as a 50% reduction of symptoms on the HRSD-6. In an intention-to-treat analysis (Table 3), the proportion of responders was 33.3% in the triple chronotherapy group, compared with 16.2% in the control group. This stayed relatively steady up to week 26, with 35.9% responders in the triple chronotherapy group, compared with 13.9% in the control group. In a per protocol analysis, the proportion of responders was 52% in the triple chronotherapy, compared with 18% in the control group at week 1 (Table 4). This gradually increased up to week 26, with 70% achieving a response in the triple chronotherapy group, compared with 22% in the control group. Of note is that 9/20 (45%) achieved a response in the first week in the triple chronotherapy group and this was maintained from week 1 onwards (supplementary Table 2). A further 6 out of 20 (30%) achieved a response from weeks 2 to 8 (when receiving bright-light therapy).

**Table 3:** Percentage achieving response by intention to treat analysis on the HRSD-6

	Triple chronotherapy		Control group	
	*n*/*N*	%	*n*/*N*	%
Week 1	13/39	33.3	7/43	16.2
Week 2	13/39	33.3	6/43	13.9
Week 4	13/39	33.3	10/43	23.3
Week 8	13/39	33.3	4/43	9.3
Week 26	14/39	35.9	6/43	13.9

HRSD-6, 6-item Hamilton Rating Scale for Depression.

**Table 4:** Percentage achieving response by per protocol analysis on the HRSD-6

	Triple chronotherapy		Control group	
	*n*/*N*	%	*n*/*N*	%
Week 1	13/25	52	7/37	18
Week 2	13/25	52	6/37	16
Week 4	13/24	54	10/33	30
Week 8	13/23	57	4/31	13
Week 26	14/20	70	6/27	22

HRSD-6, 6-item Hamilton Rating Scale for Depression.

Supplementary Table 1 shows the odds ratio for the Clinical Global Impression – Improvement scale (CGI-I), which, like the HRSD-6 and QIDS-SR, found a significant difference at weeks 1, 8 and 26. There was a large effect size of Cohen's *d* = 0.8 on the HRSD-6 at week 1, increasing to *d* = 1.30 at week 26 in a per protocol analysis (supplementary Table 3). The effect size was comparatively smaller for the QIDS-SR (*d* = 1.0 at week 26) and the PSWQ (*d* = 1.06) and slightly smaller for the RRS (*d* = 0.86), PSQI (*d* = 0.54) and EQ-5D (*d* = 0.87).

Information regarding other treatments received were collected at 26-week follow-up. There was no significant difference between groups for the number who had been receiving a psychological therapy (5/24, 21%) in the triple chronotherapy group and in the control group (4/34, 12%; χ^2^(57) = 0.36. There was also no significant difference between the number taking antidepressant medication (9/24, 37%) in the triple chronotherapy group and the sleep control group (8/34, 24%; χ^2^(57) = 0.86).

There were no reported side-effects from either intervention.

### Systematic measures of bias

There was no difference between the groups in the proportion receiving either a psychological therapy or taking medication (Table 1). We calculated fluoxetine equivalents^21^ and found no significant difference in mean fluoxetine equivalent dose between the groups (*t* = 0.14, *P* = 0.89). There was no significant difference between the groups in the rating of the credibility of the treatment (*t* = −0.739, *P* = 0.23) and the expectation for benefit (*t* = 0.44, *P* = 0.33).

## Discussion

We were able to recruit a sufficient number of participants who adhered to the treatment and therefore demonstrated the feasibility of delivering triple chronotherapy in a trial involving out-patients with depression. The potential benefits of triple chronotherapy in ensuring a rapid improvement in mood would be to prevent in-patient admission and the risk of suicide or reduce the use of other resources. A strength of the study was that there was a strong control group. Both treatments were rated as equally credible and had the same expectation for change. Some participants dropped out after they were aware of their allocated intervention group. Drop-out may not therefore have occurred at random. Despite a higher drop-out in the triple chronotherapy group, people who dropped out generally had milder depression scores at baseline. If more people dropped out of the triple chronotherapy group, then it would follow that those who remained would be more depressed. This is the reverse of what was found and supports the apparent benefits. In the triple chronotherapy group, 8 out of 39 (21%) reported difficulty with time commitments or life circumstances (including moving away, having caring responsibilities and work-related issues) that would prevent them from receiving the sleep deprivation, compared with none in the sleep control group. Two were no longer eligible owing to significant self-reported spontaneous remission of depressive symptoms. All previous trials have been conducted in in-patients, where such concerns do not occur. The drop-out of these ten participants may be partly related to the logistics of the trial as the treatment was commenced once a month (to ensure there was a group of between 2 and 4 patients for one member of staff to supervise). Future trials in out-patients could be improved by randomising participants only when they have confirmed that they can comply with the trial protocol (for example sleep deprivation on a planned day) and increasing the frequency of delivering the treatment.

The treatment, when received, was acceptable and feasible. Participants were able to adhere to the sleep deprivation with the support of the occupational therapist and did not nap during the first night. Only 2 out of the 18 (11.1%) in the triple chronotherapy group who kept sleep diaries were not able to adhere to the plan of phase advance after sleep deprivation. It was encouraging that both treatments were rated as equally credible and had the same expectation for change. Our study was a feasibility and pilot study and therefore the reported secondary outcomes are exploratory. Our findings are consistent with 4 small RCTs and 12 case series;^10^ 45% achieved response and an effect size of *d* = 0.8 after 1 week for triple chronotherapy compared with a control treatment. Triple chronotherapy has significant promise as an effective and rapid treatment for depression and that can be delivered to out-patients.

All the other secondary outcomes, including the self-report scale for depression, were in the same direction. Sleep did not significantly improve until week 2. Five out of twenty (25%) in the triple chronotherapy group achieved response from week 2 to 8, which suggests that the bright-light therapy may have an independent beneficial effect in 25% of participants. Martiny et al^22^ also found that bright-light therapy maintained response in early responders to sleep deprivation. Previous research has shown that bright-light therapy can be beneficial in non-seasonal depression. Previous meta-analyses of studies of bright-light therapy have been difficult to interpret because of the heterogeneity, with many studies conducted over 1–2 weeks.^23,24^ We are not able from this small study to determine moderators or mechanisms for change. Rumination is often associated with chronic depression.^25^ Outcomes on the Ruminative Response Scale were not significant until week 26, suggesting that triple chronotherapy may be less effective for people who are very self-critical and are ruminators.

### Limitations

This was primarily a feasibility and pilot study to determine adherence and rate of recruitment. Caution should therefore be applied, as any RCT with small numbers is always at risk of overestimating effect size and rate of response. However, our results are consistent with previous studies and meta-analysis in in-patients. To explore the secondary outcomes the end-point was the end of week 1. Thus, we wanted to ensure that both groups had a treatment in the first week that had an equal credibility and expectation rating (which we achieved). The continuation phase suggests that bright-light therapy may have added benefits. However, after week 1 the control group only had advice on sleep hygiene and treatment as usual. Future studies should control for the continuation phase. We did not conduct a systematic enquiry for possible side-effects, but none was reported. Future research is required to document formally the severity of any side-effects and their duration.

We did not explore predictors of response because of the small sample size. There are areas for improvement in a larger trial and one can improve the rates of drop-out after randomisation by better logistics in starting the treatment. We attempted to collect objective data on sleep and activity but were let down by the accuracy of the tracker. This is a recognised limitation of commercial trackers.^26^ Our participants may have exaggerated their compliance with advancing the timing of their sleep or with sleep hygiene. We did not collect any measure of light therapy adherence during the 6 months. However, there are newer actigraphs that also have a light meter and in future research, it should be possible to collect information on level of exposure to light during the treatment protocol.

This was a clinical effectiveness study and so we included participants typical of a primary care service who were taking antidepressants or receiving psychotherapy, which increases the heterogeneity of treatments received. However, there was no significant difference between the groups in terms of treatments received and importantly the credibility of the interventions and expectations. Restricting the study to participants who were not receiving any current active treatment would have meant a significantly slower recruitment rate.

### Generalisability

Our participants were out-patients mainly recruited from primary care and were not a population with severe or treatment-refractory depression. Thus, the ability to recruit and for participants to adhere to the treatment cannot be generalised to out-patients in secondary or tertiary care. Future work may also consider whether recruitment should include people from different demographic areas to ensure that findings are generalisable to a wider population.

### Interpretation

A larger definitive RCT to evaluate the clinical and cost-effectiveness of triple chronotherapy in the out-patient treatment of depression is now required.

## Data Availability

The data that support the findings of this study are available on request from the corresponding author.

## Supplementary material

For supplementary material accompanying this paper visit http://dx.doi.org/10.1192/bjo.2021.1044. click here to view supplementary material
