# Out-patient triple chronotherapy for the rapid treatment and maintenance of response in depression: Feasibility and pilot randomised controlled trial – ADDENDUM

**DOI:** 10.1192/bjo.2023.566

**Published:** 2023-11-03

**Authors:** David Veale, Marc Serfaty, Clara Humpston, Adriana Papageorgiou, Sarah Markham, John Hodsoll, Allan H Young

**Keywords:** Depressive disorders, randomised controlled trial, out-patient treatment, outcome studies, rehabilitation

The authors wish to add to the method in the previously published article. The method should read:

In a single blind trial, 82 participants were randomised to either triple chronotherapy or a control intervention. **Twelve additional participants were mistakenly randomised after screening. They were lost and had not consented to participate. The mistake occurred through human error as they should not have been randomised. No further data after screening were collected. There is no change to the analysis.** The primary outcome was the number of participants recruited per month and adherence to the protocol. Secondary outcomes included the 6-item Hamilton Rating Scale for Depression (HRSD-6) at 1 week. Timings of observer ratings were baseline and 1, 2, 4, 8 and 26 weeks after randomisation.
