# Supplementary material for: Out-patient triple chronotherapy for the rapid treatment and maintenance of response in depression: feasibility and pilot randomised controlled trial
Source: BJPsych Open. 2021 Nov 24;7(6):e220. doi: 10.1192/bjo.2021.1044 (PMC8693902; doi:10.1192/bjo.2021.1044)
Supplement: Supplementary file 1 [file S2056472421010449sup001.zip › S2056472421010449sup008.docx]

**Supplementary Table 1: Clinical Global Improvement across time: Odds Ratio with 95% Confidence Interval**

|  | Week 1 | Week 2 | Week 4 | Week 8 | Week 26 |
| --- | --- | --- | --- | --- | --- |
| Odds Ratio  (95% CI) | 5.39  (1.31, 22.18) | 2.79  (0.68, 11.39) | 4.2  (0.96, 18.29) | 20.33  (4.4, 93.87) | 24.69  (4.82, 126.38) |
| z | 2.3 | 1.4 | 1.9 | 3.9 | 3..8 |
| p | 0.019 | 0.154 | 0.056 | <0.001 | <0.001 |

**Supplementary Materials Table 2: Response maintained from Week 1 through to 26 on HAM-D6 by per protocol analysis**

|  | Triple Chronotherapy | | Control Group | |
| --- | --- | --- | --- | --- |
|  | n/ Total | % | n/ Total | % |
| Responded & maintained from 1^st^ week | 9/20 | 45% | 2/27 | 7.4% |
| Responded & maintained from 2^nd^ week | 1/20 | 5% | 0/27 | 0% |
| Responded & maintained from 4^th^ week | 1/20 | 5% | 2/27 | 7.4% |
| Responded & maintained from 8^th^ week | 3/20 | 15% | 0/27 | 0% |
| Response achieved at 26 weeks | 0/20 | 0% | 2/27 | 7.4% |
| No response at 26 weeks | 6/20 | 30% | 21/27 | 77.8% |

HAM-D6, Hamilton Rating Scale for Depression (6 items).

**Supplementary Materials Table 3: Effect Size and 95% Confidence Interval for differences between the groups by per protocol analysis**

| Outcome measure | Triple Chronotherapy | | | Control group | | | Cohen’s d | Confidence Interval for effect size | |
| --- | --- | --- | --- | --- | --- | --- | --- | --- | --- |
|  | **M** | **n** | **SD** | **M** | **n** | **SD** |  | **Lower** | **Upper** |
| HAM-D6 (Baseline) | 11.62 | 26 | 1.6 | 12.08 | 37 | 1.71 | -0.276 | -0.779 | 0.229 |
| HAM-D6 (Wk1) | 5.24 | 25 | 3.71 | 7.86 | 37 | 2.92 | -0.804 | -1.328 | -0.273 |
| HAM-D6 (Wk2) | 5.04 | 25 | 3.84 | 7.27 | 37 | 3.23 | -0.640 | -1.157 | -0.117 |
| HAM-D6 (Wk4) | 4.58 | 24 | 3.64 | 7.03 | 33 | 3.57 | -0.681 | -1.219 | -0.137 |
| HAM-D6 (Wk8) | 4.43 | 23 | 2.71 | 8.67 | 31 | 3.28 | -1.389 | -1.985 | -0.782 |
| HAM-D6 (Wk26) | 3.35 | 20 | 3.67 | 8.19 | 27 | 3.72 | -1.308 | -1.94 | -0.664 |
|  |  |  |  |  |  |  |  |  |  |
| QIDS (Baseline) | 16.23 | 26 | 2.64 | 16.08 | 37 | 3.75 | 0.045 | -0.457 | 0.546 |
| QIDS (Wk1) | 7.09 | 23 | 4.19 | 11.49 | 35 | 5.53 | -0.872 | -1.419 | -0.318 |
| QIDS (Wk2) | 8.57 | 23 | 4.63 | 11.06 | 34 | 5.09 | -0.507 | -1.042 | 0.033 |
| QIDS (Wk4) | 7.17 | 23 | 4.22 | 10.21 | 32 | 5.17 | -0.634 | -1.180 | -0.082 |
| QIDS (Wk8) | 7.45 | 22 | 4.01 | 12.38 | 31 | 5.01 | -1.066 | -1.646 | -0.477 |
| QIDS (Wk26) | 6.68 | 19 | 4.41 | 12.22 | 27 | 6.15 | -1.006 | -1.624 | -0.378 |
|  |  |  |  |  |  |  |  |  |  |
| PSWQ (Baseline) | 17.31 | 26 | 4.77 | 19.89 | 37 | 3.51 | -0.633 | -1.145 | -0.117 |
| PSWQ (Wk1) | 16.3 | 23 | 4.5 | 19.26 | 35 | 3.74 | -0.730 | -1.270 | -0.184 |
| PSWQ (Wk2) | 15.7 | 23 | 4.58 | 18.38 | 34 | 4.57 | -0.586 | -1.124 | -0.043 |
| PSWQ (Wk4) | 15.69 | 23 | 4.31 | 18.25 | 32 | 4.53 | -0.577 | -1.121 | -0.027 |
| PSWQ (Wk8) | 15.18 | 22 | 4.48 | 19 | 31 | 4.19 | -0.886 | -1.455 | -0.309 |
| PSWQ (Wk26) | 14.05 | 19 | 4.36 | 18.65 | 27 | 4.32 | -1.061 | -1.683 | -0.428 |
|  |  |  |  |  |  |  |  |  |  |
| RRS (Baseline) | 9.19 | 26 | 2.4 | 10.24 | 37 | 2.89 | -0.389 | -0.894 | 0.119 |
| RRS (Wk1) | 7.39 | 23 | 2.68 | 9.6 | 35 | 2.85 | -0.794 | -1.337 | -0.244 |
| RRS (Wk2) | 6.96 | 23 | 3.32 | 8.41 | 34 | 3.59 | -0.416 | -0.949 | 0.121 |
| RRS (Wk4) | 6.6 | 23 | 3.28 | 7.87 | 32 | 3.5 | -0.372 | -0.911 | 0.170 |
| RRS (Wk8) | 6.73 | 22 | 3.03 | 8.06 | 31 | 3.16 | -0.428 | -0.979 | 0.127 |
| RRS (Wk26) | 5.37 | 19 | 3.62 | 8.52 | 27 | 3.7 | -0.859 | -1.468 | -0.241 |
|  |  |  |  |  |  |  |  |  |  |
| PSQI (Baseline) | 11 | 26 | 3.95 | 11.35 | 37 | 3.07 | -0.101 | -0.603 | 0.401 |
| PSQI (Wk1) | 8.09 | 23 | 3.97 | 9.5 | 34 | 3.6 | -0.376 | -0.908 | 0.160 |
| PSQI (Wk2) | 6.91 | 23 | 4.02 | 9.29 | 34 | 3.51 | -0.639 | -1.179 | -0.094 |
| PSQI (Wk4) | 7.04 | 23 | 3.18 | 9.34 | 32 | 3.14 | -0.729 | -1.279 | -0.172 |
| PSQI (Wk8) | 7.36 | 22 | 3.46 | 9.74 | 31 | 3.29 | -0.708 | -1.268 | -0.142 |
| PSQI (Wk26) | 6.95 | 19 | 3.63 | 9 | 27 | 3.85 | -0.545 | -1.140 | 0.056 |
|  |  |  |  |  |  |  |  |  |  |
| EuroQoL (Baseline) | 50.38 | 26 | 20.34 | 48.38 | 37 | 24.55 | 0.087 | -0.415 | 0.589 |
| EuroQoL (Wk1) | 62.17 | 23 | 13.8 | 55 | 35 | 19.29 | 0.413 | -0.120 | 0.943 |
| EuroQoL (Wk2) | 65 | 23 | 19.54 | 55.15 | 34 | 19.68 | 0.502 | -0.038 | 1.037 |
| EuroQoL (Wk4) | 65.08 | 23 | 15.78 | 59.21 | 32 | 22.68 | 0.292 | -0.248 | 0.829 |
| EuroQoL (Wk8) | 64.5 | 22 | 18.64 | 56.13 | 31 | 21.2 | 0.415 | -0.140 | 0.965 |
| EuroQoL (Wk26) | 70.89 | 19 | 12.55 | 54.41 | 27 | 22.36 | 0.869 | 0.250 | 1.478 |

HAM-D6, Hamilton Rating Scale for Depression (6 items); QIDS, Quick Inventory of Depressive Symptomatology; PSWQ, The Penn State Worry Questionnaire; RRS, Ruminative Response Scale; PSQI, Pittsburgh Sleep Quality Index.

**Supplementary Table 4: Frequency of missing HAM-D6 outcomes by treatment group.**

| Week | Triple Chronotherapy group (N = 26) | Control group  (N =37) | All |
| --- | --- | --- | --- |
| 1 | 1 (4%) | 0 (0%) | 1 (2%) |
| 2 | 1 (4%) | 0 (0%) | 1 (2%) |
| 4 | 2 (8%) | 4 (11%) | 6 (10%) |
| 8 | 3 (12%) | 6 (16%) | 9 (14%) |
| 26 | 6 (23%) | 10 (27%) | 16 (25%) |

|  | 09:00 to 11:00 | 11:00 to 13:00 | 13:00 to 15:00 | | 15:00 to 17:00 | | 17:00 to 19:00 | | 19:00 to 21:00 | | 21:00 to 23:00 | 23:00 to 01:00 | 01:00 to 03:00 | 03:00 to 05:00 | 05:00 to 07:00 | 07:00 to 09:00 | |
| --- | --- | --- | --- | --- | --- | --- | --- | --- | --- | --- | --- | --- | --- | --- | --- | --- | --- |
| Day 0 |  |  |  | |  | |  | |  | |  |  |  |  |  |  | |
| Day 1 |  |  |  | |  | |  | |  | |  |  |  |  |  |  | |
| Day 2 |  |  |  |  | | |  | |  | |  |  |  |  |  |  |  |
| Day 3 |  |  |  | |  |  | | |  | |  |  |  |  |  |  |  |
| Day 4 |  |  |  | |  | |  |  | | |  |  |  |  |  |  |  |
| Day 5 |  |  |  | |  | |  | |  |  | |  |  |  |  |  |  |
| Day 6 |  |  |  | |  | |  | |  | |  |  |  |  |  |  |  |
| Day 7 |  |  |  | |  | |  | |  | |  |  |  |  |  |  |  |
| Day 8 onwards |  |  |  | |  | |  | |  | |  |  |  |  |  |  |  |
| Week 26 |  |  |  | |  | |  | |  | |  |  |  |  |  |  |  |

**Supplementary Figure 1: Triple Chronotherapy protocol**

| Wake | Sleep | Bright Light | Amber Glasses |
| --- | --- | --- | --- |
|  |  |  |  |

**Supplementary Figure 2 - Data points for HAM-D6 at each time point for treatment group**

**Supplementary Figure 3 - Data points for HAM-D6 at each time point for control group**

**Supplementary Figure 4 – HAM-D6 mean (Mean and SD)**
